# Tracheomalacia following a total thyroidectomy in a patient with a large non-toxic goiter: A case report

**DOI:** 10.1016/j.ijscr.2023.109211

**Published:** 2024-01-03

**Authors:** Widyanti Soewoto, Meirisa Ardianti

**Affiliations:** a Surgical Oncology Department, Medical Faculty of Universitas Sebelas Maret, Dr. Moewardi Hospital, Surakarta, Indonesia; b Surgery Department, Medical Faculty of Universitas Sebelas Maret, Dr. Moewardi Hospital, Surakarta, Indonesia

**Keywords:** Non-toxic colloid goiter, Thyroidectomy, Tracheomalacia

## Abstract

**Introduction:**

Nontoxic nodular goiter is one of the most prevalent thyroid conditions worldwide. Thyroidectomy for large goiters has a relatively high risk of postoperative airway obstruction, with tracheomalacia being one of the potential complications.

**Case report:**

A 61-year-old woman complained of a lump in her neck for 45 years. The node is progressively enlarged, but she did not experience any breathing difficulty, hoarseness, or pain while swallowing. A total thyroidectomy was then performed. The histopathologic examination revealed colloid goiter. During the procedure, evaluation of the trachea revealed a tracheomalacia, so a tracheotomy was then performed on the patient. After a follow-up period of three months, the patient was no longer experiencing tracheomalacia, and the tracheostomy was successfully closed.

**Discussion:**

Surgery has been considered an acceptable approach for managing non-toxic goiter. The most common indications are compressive symptoms, substernal extension, inability to control hyperthyroidism through medication, and a suspicion of malignancy. However, thyroidectomy for large goiter carries a relatively high risk of postoperative respiratory obstruction. Diagnosing tracheomalacia can be challenging and often relies on bronchoscopy to assess the airway and observe the collapse of cartilage and membranes. Acquired tracheomalacia can be managed through internal or external stenting or tracheostomy.

**Conclusion:**

Total thyroidectomy has been recommended as a suitable procedure for non-toxic and toxic multinodular goiter. Tracheomalacia may occur following thyroidectomy in patients with thyroid enlargement. Tracheostomy effectively manages tracheomalacia by creating a channel across the malacia's focal segment, restoring the airway's patency.

## Highlights

• Thyroidectomy is a beneficial treatment for non-toxic nodular goiter.
• Patients with large goiters were at risk of developing tracheomalacia after thyroidectomy.
• Definitive method for diagnosing tracheomalacia is through bronchoscopy.

## 1 Introduction

Non-toxic nodular goiter is a common thyroid disorder that is prevalent worldwide and can sometimes negatively affect health and well-being. It is characterized by the development of nodules in the thyroid gland, resulting from the growth of new follicles from monoclonal or polyclonal proliferation of thyrocytes [1]. Studies have linked iodine deficiency with 5–10 % of goiter cases. The age range with the highest occurrence of goiter is between 35 and 50 years, with a female-to-male ratio of 3:1 [2].

Surgery has been considered an acceptable approach for managing non-toxic multinodular goiter and has a relatively low recurrence rate [2]. However, thyroidectomy for large goiters carries a relatively high risk of postoperative respiratory obstruction, such as tracheomalacia [3]. Tracheomalacia is a rare yet potentially life-threatening complication of thyroidectomy. Post-thyroidectomy tracheomalacia is a respiratory disorder that arises after thyroidectomy, resulting from prolonged tracheal compression due to the large size of the thyroid gland.

Conversely, the authors reported an incidence from 0 % to 5.3 % [4]. Therefore, this condition must be understood better and considered infrequent [5]. In this case report, we present a case of tracheomalacia following total thyroidectomy in a patient with a sizeable, non-toxic goiter.

## 2 Presentation of case

Report a case according to SCARE criteria [5]. A 61-year-old woman residing in the endemic area has complained of a lump in her neck since she was 45 years old. The lump moves when swallowing is springy with clear boundaries and is not painful. No other nodes are present. She has no shortness of breath, hoarseness, or painful swallowing symptoms. The bulge appeared to grow more extensive within the past eight months, and she experienced signs of hyperthyroidism.

**Fig. 1:**
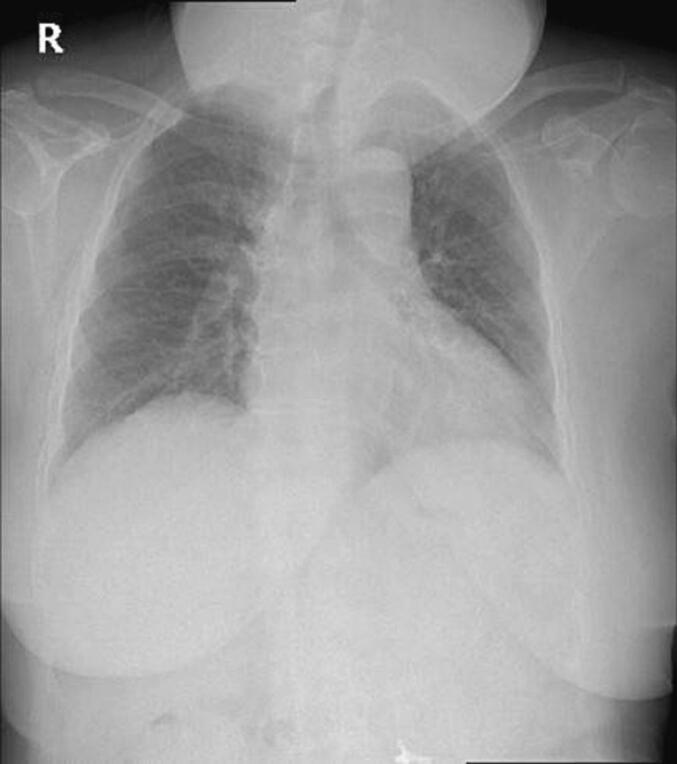
Chest X-ray examination.

The patient's general condition revealed no abnormalities on physical examination, vital signs were within normal range, and Karnosky scored 90 %. Further observation revealed the thyroid gland size was 22 × 13 × 13 cm, with a bumpy surface. The skin color is the same as the surrounding skin, and the boundaries are clear, with no tenderness on palpation. Chest X-ray examination revealed a mass in the Colli area that appeared pressing on the trachea; no cardiomegaly was found, and both lung fields appeared normal (Fig. 1). Ultrasonography (USG) examination and CT scan showed multiple masses in the thyroid without microcalcifications.

**Fig. 2:**
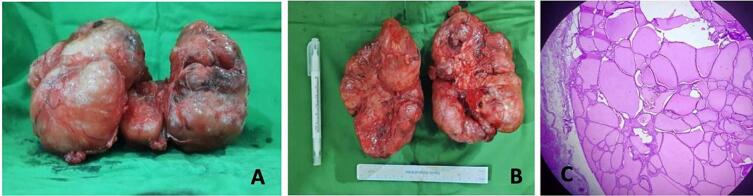
A histopathological study revealed a colloid goiter.

We performed a total thyroidectomy on the patient, and we found the right thyroid was 13 × 10 × 8 cm while the left thyroid was 13 × 9 × 10 cm in size with some complex and cystic parts (Fig. 2A–B). The recurrent and superior laryngeal nerves are intact. During the surgery, it was found that the mass was pressing against the trachea. After the thyroid could be removed, the trachea appeared intact and palpable soft.

Following the total thyroidectomy and before the extubation, the trachea was assessed by laryngoscopy and showed narrowing of the tracheal lumen with loss of semicircular shape and bulging of the posterior membrane wall. Tracheomalacia was identified, leading to the need for a tracheostomy.

**Fig. 3:**
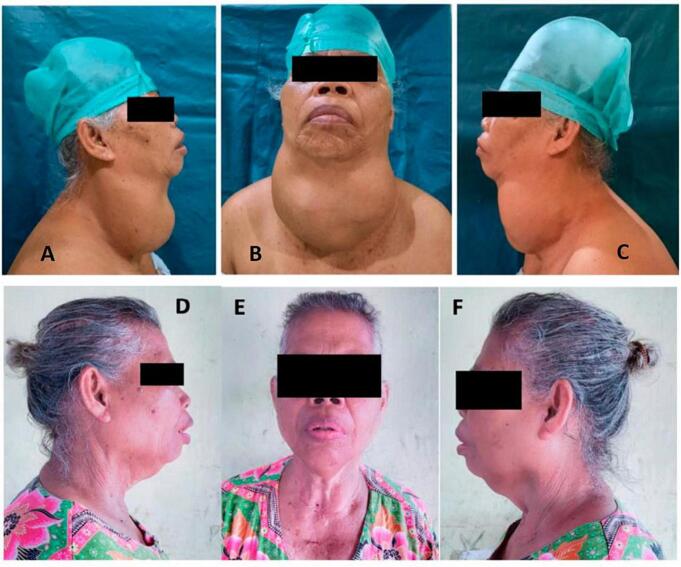
A–C. 61-year-old woman with a bumpy and mobile lump on the thyroid, 25 × 15 × 15 in size, with clear boundaries. D–F. Patient's clinical condition on two-year follow-up.

The patient's overall condition was stable after the surgery; compos mentis with typical vital signs and oxygen saturation levels of 99 % with the assistance of 3 L of oxygen per minute through the tracheostomy. Twenty-four hours after the surgery, the patient had no hoarseness, and the test results showed hypocalcemia (1.8 mmol/L), and the calcium levels were corrected. After the 3rd day, the calcium level returned to normal. On the second day after the surgery, the patient's thyroid function test results showed low levels of TSH (<0.05 μIU/mL) and normal FT4 levels (30.19 pmol/L). The patient's thyroid function tests and calcium were repeated one week after surgery. The results showed low levels of TSH (<0.05 μIU/mL), free FT4 (4.60 pmol/L), and calcium levels are normal. As a result, the patient was prescribed Euthyrox 100 μg (Fig. 3).

The histopathology results revealed that thyroid tissue with glandular follicular hyperplasia had colloid cells with uniform nuclei, no images of capsule invasion or vascular invasion, and no symptoms of malignancy (Fig. 2C).

Ten days after surgery, the patient was discharged with a tracheostomy still attached. The patient is advised to check his condition regularly and to do respiratory physiotherapy periodically in the hospital. In addition, patients also practice opening and closing tracheostomy independently; this is done by patients approximately one month after surgery. After three months, the patient's clinical evaluation showed good progress, and the patient could breathe spontaneously with the tracheostomy closed. Bronchoscopy to assess the condition of the trachea was not done due to limited patient insurance, so it was decided to close the tracheostomy at the surgical oncology clinic.

Two years after surgery, an evaluation was carried out; postoperative ultrasound results showed no recurrent masses in the thyroid. The patient's overall condition is good; she can do her usual activities—no respiratory distress or clinical symptoms of hypocalcemia.

## 3 Discussion

Thyroid nodules are common in medical practice, and while most are benign, about 5 % of thyroid nodules are malignant. The prevalence of thyroid nodules is higher in women and areas with iodine deficiency and increases with age. Nontoxic goiter is the most common thyroid disorder worldwide, characterized by either localized or diffuse enlargement of the thyroid with normal thyroid function and changes in the follicles' structure and function. [2,7,8]. Surgery is a common approach for managing goiter [2]. The main reasons for surgery include experiencing symptoms of compression (such as difficulty swallowing or breathing), the goiter extending below the sternum, hyperthyroidism that doesn't respond to medication, and suspicion of cancer [[9], [10], [11]]. Different types of surgical procedures are available, such as total thyroidectomy, near-total thyroidectomy, subtotal thyroidectomy, isthmolobectomy, or lobectomy [11].

In this particular case, the MSCT (Multislice Computerized Tomography) scan revealed an abnormal thyroid image, and during the surgery, it was confirmed that no normal thyroid tissue was present. Therefore, a total thyroidectomy was decided upon. Although thyroidectomy has its benefits in reducing the recurrence rates, it is essential to note that performing this procedure on patients with large goiters comes with a relatively high risk of postoperative respiratory obstruction. This obstruction may be severe enough to require a tracheostomy [3]. The causes of this obstruction in patients with enlarged thyroids include local bleeding, bilateral recurrent nerve damage, swelling of the larynx, and tracheomalacia [13,19].

In our case, during the surgery, a mass was discovered that was exerting pressure on and causing displacement of the trachea. After the complete removal of the thyroid gland, tracheomalacia was identified, leading to the decision to perform a tracheostomy. The patient was monitored for three months after the procedure and showed no signs of tracheomalacia.

Tracheomalacia is characterized by weakness in the tracheal wall due to softening of the supporting cartilage and hypotonia of myoelastic elements [13,15]. Causes of acquired tracheomalacia include trauma, surgery, chronic irritation, infection, mechanical changes, and malignant infiltration [17]. In the past, it was believed that patients with large goiters were at risk of developing tracheomalacia after thyroidectomy. This condition is typically a result of prolonged compression on the trachea, leading to the collapse of >50 % of the airway diameter. [14,18].

The incidence of tracheomalacia after thyroidectomy is estimated to be between 0.8 % and 5.8 %. Several studies have examined the risk of tracheomalacia as a respiratory complication following thyroidectomy [15,19]. A prospective study conducted by Rahim et al. reported that 32 patients (31 %) experienced postoperative respiratory complications, and 13 of them required tracheostomy, and tracheomalacia was the most common indication (5 patients) [3]. In a cross-sectional and retrospective study conducted by Ayandipo et al. between 2001 and 2005, it was found that out of 507 patients who underwent thyroidectomy, the majority were women (86 %). Tracheomalacia was observed in 27 patients (5.3 %), and two of these patients died due to respiratory problems [18].

In a study conducted by Valizadeh et al., it was found that out of 1236 patients who underwent thyroidectomy, there were no cases of tracheomalacia [4]. Similarly, Bennett et al. reviewed 12 articles and discovered that out of a total of 1969 cases of thyroidectomy, there was no tracheomalacia or collapse of the trachea and airway [19].

Acquired tracheomalacia can be managed with internal/external stenting or tracheostomy. Tracheostomy is an effective method for addressing tracheomalacia as it creates a passage through the affected segment, opening the airway [6,17]. In cases where patients struggle to maintain adequate oxygen saturation, intubation for an extended period may be necessary. If difficulties with extubation persist after approximately two weeks, tracheostomy is typically advised. The inflammatory response triggered by the insertion of a tracheostomy or endotracheal tube leads to the stiffening of the tracheal walls, thereby preventing expiratory collapse [14,15].

Sabertnam et al. recommend performing intraoperative tracheostomy if there is clear softening of the trachea, and it is preferable to perform intraoperative tracheostomy because it is easier to visualize the part of the trachea, as portrayed in this case. In contrast to prolonged intubation, tracheostomy induces fibrosis around the delicate trachea, leading to a prompt recovery from tracheomalacia. Typically, tracheostomy patients have their tracheostomy tube removed within a week [14,17]. On the other hand, tracheopexy is selected for cases of short-segment lateral tracheomalacia. This technique prevents posterior collapse and maintains an unobstructed airway [14].

## 4 Conclusion

Thyroidectomy has been widely accepted as an effective treatment for goiter, particularly in cases where the goiter is non-toxic. Total thyroidectomy is often recommended due to its low recurrence rate. However, it is essential to note that tracheomalacia, a rare complication, can occur following thyroidectomy in patients with enlarged thyroids. The definitive method for diagnosing tracheomalacia is through bronchoscopy, which allows for the identification of cartilage collapse and tracheal membrane collapse. Tracheostomy effectively treats tracheomalacia by creating a pathway through the affected airway segment, thus restoring proper airflow.

## Ethical consent

It is not required for case reports in our hospital. Single-case words are excluded from ethical approval at our institution.

## Patient consent

Written informed consent was obtained from the patient for publication and any accompanying images. A copy of the written consent is available for review by the Editor-in-Chief of this journal on request.

## Funding statement

This research received no specific grant from funding agencies in the public, commercial, or not-for-profit sectors.

## Credit authorship contribution statement

Widyanti Soewoto: study design, data collection/analysis, writing the paper.

Meirisa Ardianti: study design, data collection/analysis, writing the essay.

## Guarantor

Widyanti Soewoto.

## Research registration number

1. Name of the registry: Widyanti Soewoto.

2. Unique identifying number or registration ID: https://orcid.org/0000-0003-2513-5801.

## Conflicts of interest

All authors declare that they have no conflicts of interest.
